# Funding Trends and Gender Disparities in Tanzanian Biomedical Research: Insights From Global Health Funding Databases

**DOI:** 10.7759/cureus.94912

**Published:** 2025-10-19

**Authors:** Raphael Z Sangeda, Siana Nkya, Upendo Masamu, Rehema C Mallya, Magdalena Lyimo, Bruno P Mmbando, Nahya Salim

**Affiliations:** 1 Department of Pharmaceutical Microbiology, Muhimbili University of Health and Allied Sciences, Dar es Salaam, TZA; 2 Department of Biochemistry and Molecular Biology, Muhimbili University of Health and Allied Sciences, Dar es Salaam, TZA; 3 Muhimbili Sickle Cell Programme, Muhimbili University of Health and Allied Sciences, Dar es Salaam, TZA; 4 Directorate of Library Services, Muhimbili University of Health and Allied Sciences, Dar es Salaam, TZA; 5 Department of Haematology and Blood Transfusion, Muhimbili University of Health and Allied Sciences, Dar es Salaam, TZA; 6 Department of Public Health, Biostatistics and ICT, National Institute for Medical Research, Tanga, TZA; 7 Department of Paediatric and Child Health, Muhimbili University of Health and Allied Sciences, Dar es Salaam, TZA

**Keywords:** funding organizations, funding trends, gender disparities, national institutes of health (nih), research funding, research institutions, tanzania, world report

## Abstract

Background

Research funding is crucial for advancing scientific knowledge and innovation. Understanding patterns and trends in research funding, including potential gender disparities, can provide valuable insights for policymakers, funding agencies, and researchers. This study aimed to analyze the distribution of research funding in Tanzania, identify major contributors, uncover trends over the past years, and examine gender disparities among principal investigators (PIs).

Methods

Data from the National Institutes of Health (NIH) funding database and World RePORT were utilized. Data cleaning and preprocessing ensured consistency and accuracy, with funding amounts converted to common currency (USD) for analysis. Statistical and trend analyses were performed to identify key patterns, focusing on funding distribution by research organization, funding organization, fiscal year, type of record, and PI gender.

Results

A total of $218,409,329 were distributed across 438 projects led by 324 unique PIs. Male PIs accounted for 228 (70.4%), females for 91 (28.1%), and five (1.5%) were of unspecified gender. Collaborative projects comprised 374 entries ($158,121,528.60), and direct funding accounted for 64 projects ($60,287,799.96). The European and Developing Countries Clinical Trials Partnership contributed the most ($165,598,586; 75.8%), followed by the Wellcome Trust ($12,282,858; 5.6%) and the European Commission ($12,017,629; 5.5%). Funding rose steadily, peaking in 2020 and 2022 at over US $40 million. The National Institute for Medical Research received the highest total cost (US$95,453,357; 43.7%). Male PIs led more projects and received $49,038,802, compared to $22,475,127 for female PIs. In 2023, however, female PIs received more funding ($5,517,171) than male PIs ($4,354,089), reversing previous trends.

Conclusions

Research funding in Tanzania remains concentrated among a few institutions and is dominated by one major donor. Although overall funding has increased, significant gender disparities persist. The 2023 shift toward greater female PI funding is promising but requires sustained policy attention. These insights can inform equitable research and investment strategies for Tanzania.

## Introduction

Scientific research is a key driver of innovation, economic development, and improved health outcomes. In countries with limited resources, such as Tanzania, research funding plays a crucial role in building local capacity, advancing public health goals, and contributing to global scientific efforts [1-4].

Although funding can boost research productivity and strengthen institutions, access to funding is not always equal. Differences in funding levels across institutions, donor priorities, and researcher characteristics, including gender, can influence who leads research projects and which topics receive attention [5,6]. Exploring these patterns is important for fostering a research environment that is fair, inclusive, and aligned with national needs.

Despite growing attention to these issues, comprehensive data on how research funding is distributed in Tanzania remains limited. There is a need to better understand how funding varies by institution, funding source, and the gender of the principal investigators (PIs). This information can support efforts to improve equity, enhance transparency, and inform strategic research plans.

World RePORT, an interactive and open-access database developed by the National Institutes of Health (NIH), offers a valuable resource for analyzing global biomedical research investments. It captures data on projects funded by major international donors and provides information on funding organizations, recipient institutions, collaborating researchers, and project leaders. The platform also enables users to explore funding trends by location and research area [7,8].

In this study, we analyzed data from the World RePORT and NIH funding databases to examine research funding patterns in Tanzania from 2016 to 2023. Specifically, this study aims to characterise biomedical research funding patterns by identifying major funders and recipient institutions, evaluating funding trends over time, and assessing gender disparities among PIs.

This work was presented at the 13th Muhimbili University of Health and Allied Sciences (MUHAS) Scientific Conference, held on 18-19 June 2025 in Dar es Salaam, Tanzania.

## Materials and methods

Data sources

We obtained project-level funding data specific to Tanzania from the NIH funding database and the World RePORT platform [7,8]. These data include funding amounts (in USD), project titles, PIs, recipient and collaborating institutions, and associated funding agencies. Projects active between January 1, 2016, and December 31, 2023, were included; duplicate records across sources were identified and removed to ensure consistency.

Data preparation

Data cleaning involved removing duplicates, checking for missing or inconsistent values, and standardizing monetary values by converting all funding amounts to USD using annual World Bank exchange rates. Projects were categorized into direct funding or collaborative funding based on the role of Tanzanian institutions. Direct funding refers to projects in which Tanzanian organizations were the primary recipients, while collaborative funding refers to projects in which Tanzanian institutions were partners. Additional formatting included harmonizing column headers, correcting data types (e.g., fiscal year, start and end dates, funding amounts), and ensuring consistency across merged datasets.

Principal investigators were mapped to recipient organizations through a structured lookup process, allowing aggregation of funding amounts and assignment of gender attributes for further analysis.

Gender identification

The gender of PIs was inferred from first names using culturally informed naming conventions. For ambiguous cases, gender was verified through institutional websites, publication profiles, ORCID records, or other professional online sources. Gender classification was limited to binary categories (male or female) based on available information.

Data analysis and visualization

Descriptive statistical analysis was conducted using Microsoft Excel and Power BI (Microsoft Corp., USA), following a stepwise approach that included categorizing projects, aggregating funding by donor and institution, and generating time-series summaries to examine funding trends, donor distributions, and institutional allocation. Visualizations, including bar and line charts, were used to illustrate annual changes, highlight major funders and recipients, and compare direct and collaborative funding. Gender-based comparisons focused on both the number of funded projects and the total funding amounts awarded to male and female PIs.

Ethics statement

This study did not involve human participants, animal subjects, or patient data. All analyses were conducted using publicly available data from the NIH RePORTER databases. Therefore, ethical approval and informed consent were not required.

## Results

**Figure 1:**
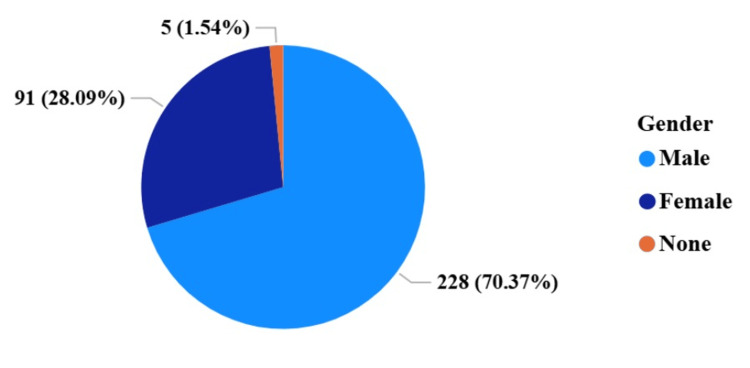
Gender distribution of principal investigators (PIs) in Tanzania’s funded biomedical research portfolio (2016–2023) Proportions of male (70.4%), female (28.1%), and unspecified (1.5%) PIs based on 324 unique investigators identified in NIH RePORTER and World RePORT.

Between 2016 and 2023, research funding in Tanzania totaled US $218,409,329 across 422 unique projects. A total of 324 PIs were identified, of whom 228 (70.4%) were male, 91 (28.1%) were female, and five (1.5%) had an unspecified gender (Figure 1).

Collaborations accounted for 374 projects, with a total funding of $158,121,528.60, while direct funding comprised 64 projects, with a total funding of $60,287,799.96 (Table 1).

**Table 1:** Funding distribution by biomedical research project type and gender of principal investigators (PIs) in Tanzania (2016–2023) Data extracted from NIH RePORTER and World RePORT. Male PIs led 273 projects ($49.0 million), female PIs led 147 projects ($22.5 million), and five were unspecified. Collaborative projects (n = 374; $158.1 million) accounted for the majority of funding compared to direct projects (n = 64; $60.3 million).

		Funding entries	Unique projects	Total funding (USD)
Gender	Not specified	381	55	146,895,399.50
	Male	1032	273	49,038,802.21
	Female	503	147	22,475,126.88
Type of funding				
	Collaboration	1723	374	158,121,528.63
	Direct	193	64	60,287,799.96

**Figure 2:**
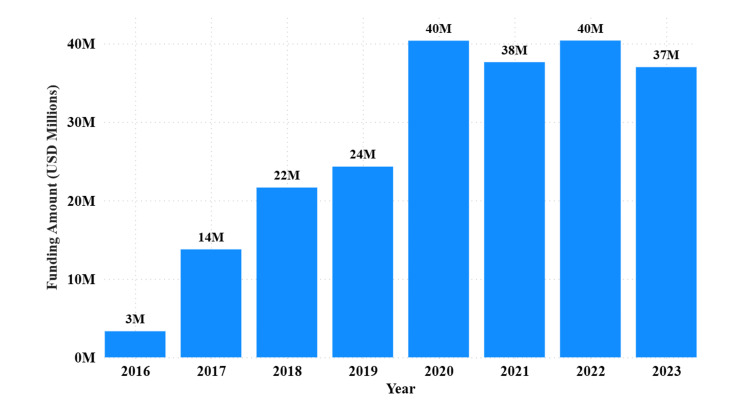
Annual biomedical research funding in Tanzania by fiscal year (2016–2023) Total annual funding amounts (USD) awarded to Tanzanian biomedical research institutions, with peaks in 2020 ($40.4 million) and 2022 ($40.4 million).

Annual funding has increased steadily from 2016 to 2023, with notable peaks in 2020 and 2022, each surpassing USD 40 million. Funding increased significantly from 2016 to 2023, with the highest funding recorded in 2020 ($40,361,911.02) and 2022 ($40,380,295.74). The detailed yearly funding amounts (Figure 2).

**Figure 3:**
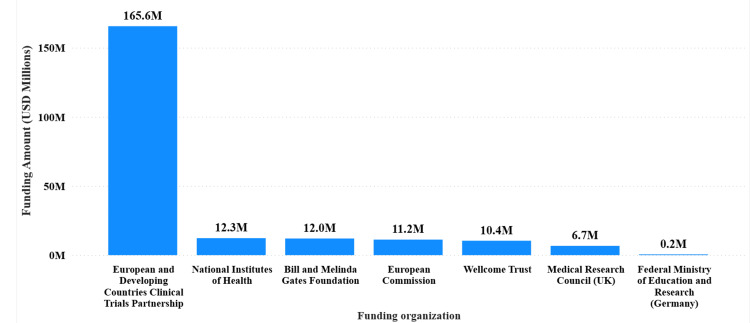
Major international donors contributing to Tanzanian biomedical research (2016–2023) Cumulative contributions from leading global funders. The European and Developing Countries Clinical Trials Partnership (EDCTP) provided the largest share ($165.6 million, 75.8%), followed by the Wellcome Trust, European Commission, NIH, and the Bill & Melinda Gates Foundation.

The European and Developing Countries Clinical Trials Partnership was the largest funder, contributing $165,598,586, accounting for 75.8% of the total funding (Figure 3). Other significant contributors included the Wellcome Trust ($12,282,858, 5.6%), the European Commission ($12,017,629, 5.5%), the National Institutes of Health ($11,226,129, 5.1%), and the Bill and Melinda Gates Foundation ($10,408,309, 4.8%).

**Figure 4:**
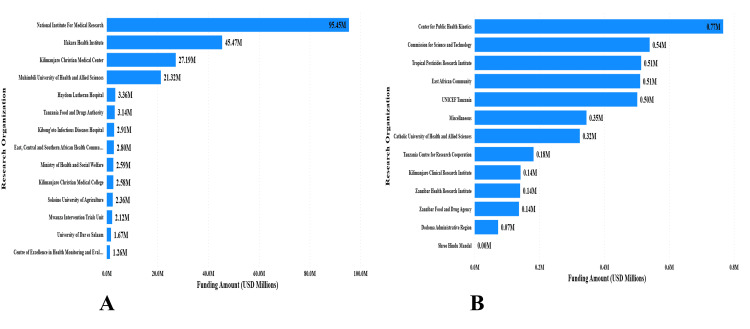
Research funding received by Tanzanian biomedical research institutions (2016–2023) Panel A: Institutions receiving ≥$1 million in cumulative funding. Panel B: Institutions receiving < $1 million. The National Institute for Medical Research (NIMR) received the largest total ($95.5 million, 43.7%), followed by the Ifakara Health Institute ($45.5 million, 20.8%) and Kilimanjaro Christian Medical Centre ($27.2 million, 12.5%).

The National Institute for Medical Research (Tanzania) received the highest funding, totaling $95,453,357, representing 43.7% of the total funding. The Ifakara Health Institute received $45,469,713 (20.8%), followed by Kilimanjaro Christian Medical Center with $27,194,631 (12.5%) (Figure 4).

**Figure 5:**
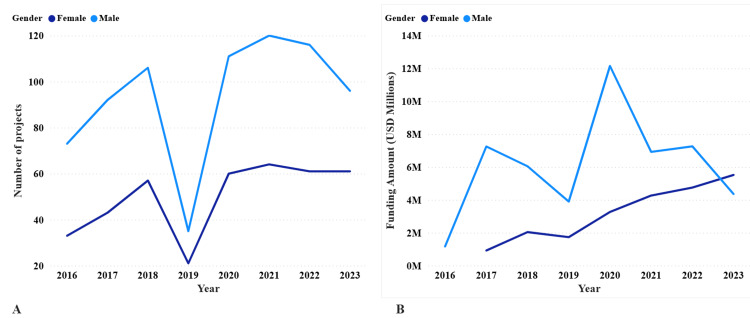
Gender differences in biomedical research funding in Tanzania (2016–2023) Panel A: Number of funded projects per year by PI gender. Panel B: Annual funding amounts (USD) awarded to male versus female PIs. Male PIs consistently led more projects and received more funding, except in 2023, when female PIs received a higher total ($5.5 million) than male PIs ($4.3 million).

Gender disparity analysis revealed that male PIs consistently led more projects and received greater funding than female PIs in most years (Figure 5A, 5B). In 2016, male PIs led 73 projects and received USD 1,160,947, whereas female PIs led 33 projects but received no recorded funding. This trend continued, with the disparity most pronounced in 2020, when male PIs led 111 projects and received USD 12,152,424, compared to 60 projects and USD 3,263,475 for female PIs. Notably, in 2023, female PIs surpassed male PIs in total funding received, with USD 5,517,171 compared to USD 4,354,089. Cumulatively, male PIs led 749 projects and received USD 49,038,802, whereas female PIs led 463 projects and received USD 22,475,127, a funding gap of USD 26,563,675 over the study period.

## Discussion

This analysis of research funding in Tanzania between 2016 and 2023 provides critical insights into funding distribution, institutional participation, and gender disparities. The findings highlight the structural patterns that influence research output and equity in access to funding.

Our analysis revealed that a large proportion of biomedical research funding in Tanzania was directed toward collaborative projects, which accounted for over 70% of the total funding. This aligns with previous findings from low- and middle-income countries (LMIC) contexts, where donors often favor collaborative research due to its potential for capacity building and resource sharing [1]. Emphasis on collaboration may reflect the perceived added value of cross-institutional expertise in addressing complex health challenges. However, as noted in studies of NIH funding in similar settings [9], heavy reliance on collaborative models may also limit direct institutional autonomy and constrain locally driven research agendas. Ensuring that collaborative projects contribute to local leadership and infrastructure development is crucial for long-term research sustainability.

The funding landscape was dominated by a few international donors, with the European and Developing Countries Clinical Trials Partnership contributing over 75% of the total funding. This high level of concentration is consistent with the trends observed in other analyses of global health research, where a small number of funding bodies exert significant influence over national research priorities [10,11]. While large-scale investments from organizations such as the Wellcome Trust, European Commission, and NIH provide vital support in resource-limited settings, overdependence on external donors may create vulnerability to shifts in global funding agendas. Recent Analyses by Hussein and Samet illustrate the real-world consequences of such dependencies, showing how abrupt policy changes-such as the US withdrawal from the WHO and cuts to USAID-disrupted critical health programs in LMICs [12]. Similar concerns have been raised in other studies, where donor-driven priorities sometimes limit the alignment of research with national health needs [13]. Policymakers in Tanzania may need to balance international engagement with increased domestic investment to ensure strategic autonomy in setting up and sustaining a national research agenda.

The upward trend in research funding from 2016 to 2023, particularly the peaks in 2020 and 2022, underscores the growing global commitment to health research in LMICs. The spike in 2020 likely reflects emergency funding responses to the COVID-19 pandemic, mirroring patterns observed in other global health systems where research priorities have shifted rapidly to address emerging threats [14,15]. Sustained year-over-year increases also suggest that donor agencies recognize the long-term value of investing in local research ecosystems. As observed in other NIH funding trend analyses [9,13] [9], such momentum is essential for strengthening scientific output and national preparedness for future public health emergencies.

The disproportionate allocation of funding to a few institutions, notably the National Institute for Medical Research (NIMR), Ifakara Health Institute, Kilimanjaro Christian Medical Center, and Muhimbili University of Health and Allied Sciences (MUHAS), reflects the institutional concentration often observed in LMICs with limited research infrastructure [11,16]. While this concentration may indicate organizational maturity, grant management capacity, and strong international collaborations, it also raises important questions regarding the equitable distribution of funding across the broader research ecosystem. Similar patterns have been observed in U.S.-based NIH funding, in which well-established institutions often dominate grant portfolios [9]. To enhance research inclusivity and regional balance, there is a need to build the capacity of emerging institutions to effectively compete for funding and contribute meaningfully to national and global scientific efforts.

Gender disparities in research funding remain a persistent global challenge, and our analysis reflects similar trends in Tanzania. Male PIs consistently led more projects and received significantly higher funding compared to their female counterparts across most years, with the largest gap observed in 2020. This pattern mirrors findings from other NIH-related studies that report systemic underrepresentation of women in both first authorship and grant leadership roles [5,11,17]. However, the reversal observed in 2023, where female PIs received more funding than males, marks a potentially meaningful shift. This could reflect growing institutional efforts or donor-driven priorities aimed at promoting gender equity. However, the cumulative disparity over the whole study period underscores the need for sustained policy and structural interventions to ensure equal access to funding opportunities. Advancing gender equity in research leadership is not only a matter of fairness but also essential for enhancing innovation and inclusivity in scientific inquiry.

Although a detailed correlation between funding and research output, such as publications or citations, is beyond the scope of this analysis, prior work [18,19] has demonstrated a positive association between research funding and scientific productivity in Tanzania. These earlier findings indicate that higher levels of research funding are positively correlated with increased scientific output and citation impact [20]. This reinforces the importance of sustained investment in building a productive research ecosystem, particularly in resource-limited settings.

Our current findings also resonate with global NIH-related trends, such as those reported by Abraham et al. (2023) and Chaudhary et al. (2021), who consistently showed lower female representation in funded research roles [5,11]. Similarly, Mahajan et al. highlighted a growing trend toward gender parity in Tourette syndrome research, with statistical forecasts projecting an increase in female authorship by 2027 [21]. These global patterns underscore both the progress and persistent barriers women face in research leadership. In our context, the funding reversal observed in 2023, where female PIs in Tanzania received more total funding than their male counterparts, may reflect early signs of such progress. This shift could be attributed to institutional reforms or donor priorities that emphasize inclusion. Unlike studies focused on high-income settings [9,10], our findings provide LMIC-specific evidence that highlights the dual imperative of equitable funding and local capacity building. Reducing reliance on dominant institutions and fostering gender-balanced leadership may contribute to a more inclusive and sustainable research ecosystem in resource-limited settings.

Study limitations

Despite offering important insights into research funding patterns in Tanzania, this study has several limitations. First, the analysis is descriptive only and does not test whether observed differences or trends are statistically significant; some patterns may partly reflect random variation. Second, the analysis relies solely on data from the NIH RePORTER and World RePORT platforms, which do not capture all relevant funding sources, particularly from domestic, regional, or private institutions. This reliance likely biases results toward international donor activity. Third, gender classification was inferred primarily from names and cross-verified through professional sources, and was restricted to binary categories (male and female), reflecting the prevailing conventions in the available data and in the Tanzanian research context. Finally, other potentially influential factors, such as academic discipline, institutional support mechanisms, or the career stage of investigators, were not examined. The dataset reflects awarded projects only and does not capture downstream outcomes such as publications, patents, or translational impacts, which would provide a fuller picture of research productivity.

## Conclusions

This study demonstrates that research funding in Tanzania remains concentrated among a few institutions and is primarily driven by a small number of international donors. Although the overall increase in funding from 2016 to 2023 reflects a positive investment trend, persistent gender disparities in project leadership and funding allocation highlight the need for targeted strategies to promote equity. The reversal observed in 2023, with female PIs receiving more total funding than their male counterparts, signals encouraging but still fragile progress.

Beyond describing these trends, the findings highlight the importance of structural and policy responses. Strengthening domestic co-funding schemes, expanding mentorship and grant-writing support for early-career and female investigators, and embedding equity indicators into national research monitoring systems are practical steps that could accelerate progress toward a more balanced research environment. By providing these insights, the study informs both donor priorities and institutional reforms to foster a more inclusive and sustainable research ecosystem in Tanzania.

## References

[1] Alinani A, Mills B, Gause E, Vavilala MS, Lele AV (2022). National Institutes of Health clinical research funding and all-cause in-hospital traumatic brain injury-related mortality. Cureus.

[2] Said Sife A, Tandi Lwoga E (2014). Publication productivity and scholarly impact of academic librarians in Tanzania. New Libr World.

[3] Lwoga ET, Sangeda RZ, Sife AS (2017). Online visibility of pharmacy research in Tanzania: a scientometric study. Int J Pharm Pharm Sci.

[4] Lwoga ET, Sife AS (2013). Mapping the research productivity and scholarly impact of the traditional medicine scholars in tanzania : a scientometric analysis. Int J Digit Library Serv.

[5] Abraham J, Panchal K, Varshney L, Lakshmi Narayan K, Rahman S (2023). Gender disparities in first authorship in publications related to attention deficit hyperkinetic disorder (ADHD) and artificial intelligence (AI). Cureus.

[6] Asturias AM, Wague A, Feeley LA, Senter C, Pandya N, Feeley BT (2024). Gender disparities in endowed professorships within orthopaedic surgery. Cureus.

[7] (2025). NIH: World RePORT. https://worldreport.nih.gov/wrapp/.

[8] Berg EJ, Ashurst J (2019). Patterns of recent National Institutes of Health (NIH) funding in general surgery: analysis using the NIH RePORTER system. Cureus.

[9] Berg EJ, Ashurst J (2020). National Institutes of Health funding in obstetrics and gynecology: analysis of R01 grants by degree and gender. Cureus.

[10] Mutwiri G Jr, Kulanthaivelu R, Yuen J (2022). Gender differences among academic radiation oncology National Institutes of Health (NIH) funding recipients. Cureus.

[11] Chaudhary AM, Naveed S, Safdar B, Saboor S, Zeshan M, Khosa F (2021). Gender differences in research project grants and R01 grants at the National Institutes of Health. Cureus.

[12] Hussein S, Samet JM (2025). Measuring population health impact of the Trump administration's withdrawal from WHO and cuts to USAID: time to start counting. Popul Health Metr.

[13] Berg EJ, Santarelli A, Ashurst J (2021). National Institutes of Health funding in internal medicine: analysis of physicians receiving an R01 grant between 2008 and 2017. Cureus.

[14] Okobi OE, Ibanga IU, Egbujo UC, Egbuchua TO, Oranu KP, Oranika US (2023). Trends and factors associated with mortality rates of leading causes of infant death: a CDC wide-ranging online data for Epidemiologic Research (CDC WONDER) database analysis. Cureus.

[15] Sharma S, Anghole AA, Pathare SB, Nagare MR, Choubey S, Malik A (2023). Breaking barriers: investigating gender representation in the first authors of cardiovascular disease and artificial intelligence publications. Cureus.

[16] Maqsood H, Younus S, Naveed S, Chaudhary AM, Khan MT, Khosa F (2021). Sticky floor, broken ladder, and glass ceiling: gender and racial trends among neurosurgery residents. Cureus.

[17] Safdar B, Naveed S, Chaudhary AM, Saboor S, Zeshan M, Khosa F (2021). Gender disparity in grants and awards at the National Institute of Health. Cureus.

[18] Vijayakumar V, Babu HF, Karki A, Tyagi R, Macapia M, Zapata KM, Dogiparthi S (2023). Gender disparity of first authors in review article publications related to schizophrenia. Cureus.

[19] Sangeda RZ, Lwoga T (2017). Research growth and citation impact of Tanzanian scholars: a 24 years scientometric study. Int J Lib Inf Sci.

[20] Fortin JM, Currie DJ (2013). Big science vs. Little science: how scientific impact scales with funding. PLoS One.

[21] Mahajan A, K V, Dikshit N, Sandhu JK, Pallempati LL, Olivieri L (2024). Gender representation in academic publications of Tourette syndrome research: an analysis of authorship trends. Cureus.

